# Degree-day-based model to predict egg hatching of *Philaenus spumarius* (Hemiptera: Aphrophoridae), the main vector of *Xylella fastidiosa* in Europe

**DOI:** 10.1093/ee/nvad013

**Published:** 2023-04-19

**Authors:** Clara Lago, Àlex Giménez-Romero, Marina Morente, Manuel A Matías, Aránzazu Moreno, Alberto Fereres

**Affiliations:** Instituto de Ciencias Agrarias (ICA-CSIC), Serrano 115b, 28006, Madrid, Spain; Departamento de Producción Agraria, Escuela Técnica Superior de Ingeniería Agronómica, Alimentaria y de Biosistemas (ETSIAAB), Universidad Politécnica de Madrid (UPM), Avenida Puerta de Hierro, 2,4, 28040, Madrid, Spain; Instituto de Física Interdisciplinar y Sistemas Complejos (IFISC, CSIC-UIB), Campus UIB, 07122, Palma de Mallorca, Spain; Instituto de Ciencias Agrarias (ICA-CSIC), Serrano 115b, 28006, Madrid, Spain; Instituto de Física Interdisciplinar y Sistemas Complejos (IFISC, CSIC-UIB), Campus UIB, 07122, Palma de Mallorca, Spain; Instituto de Ciencias Agrarias (ICA-CSIC), Serrano 115b, 28006, Madrid, Spain; Instituto de Ciencias Agrarias (ICA-CSIC), Serrano 115b, 28006, Madrid, Spain

**Keywords:** modelling, control, vector-borne disease, integrated pest management

## Abstract

*Philaenus spumarius* L., the main vector of *Xylella fastidiosa* (Wells) in Europe, is a univoltine species that overwinters in the egg stage, and its nymphs emerge in late winter or spring. Predicting the time of egg hatching is essential for determining the precise times for deploying control strategies against insect pests. Here, we monitored *P. spumarius* eggs from oviposition to egg hatching together with the daily temperatures and relative humidities at four field locations that were located at different altitudes in central Spain. The collected data were used to build a growing degree day (GDD) model to forecast egg hatching in the Iberian Peninsula. Furthermore, the model was validated with field observations that were conducted in Spain. The model was then used as a decision-support tool to calculate the optimum timing for applying control actions against *P. spumarius*. Our results suggest that controlling nymphs at two different dates would target the highest percentages of nymphal populations present in the field. Our model represents a first step for predicting the emergence of nymphs and adopting timely control actions against *P. spumarius.* These actions could limit disease spread in areas where *X. fastidiosa* is present.

## Graphical Abstract

**Graphical Abstract:**
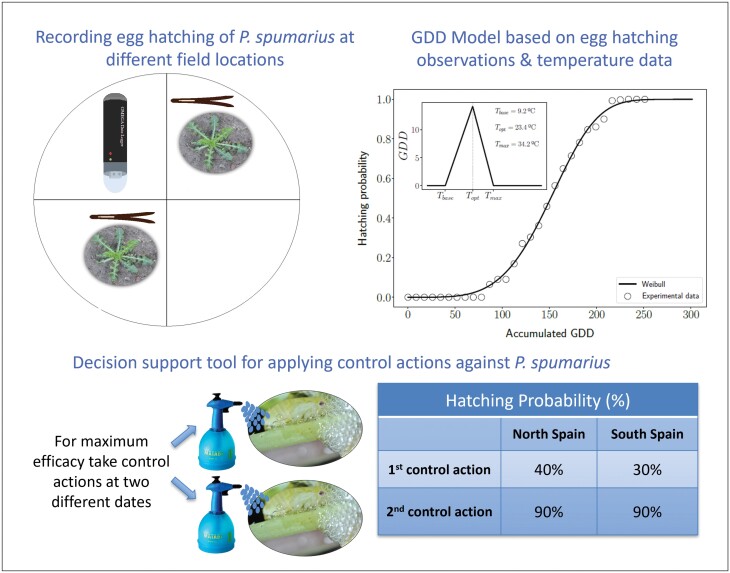

## Introduction

Predicting epidemics can be challenging, especially after the emergence of a new pathogen in a novel ecosystem. The introduction of *Xylella fastidiosa* (Wells, 1987) (Xanthomonadales: Xanthomonadaceae) in Europe and the Mediterranean basin represents a major threat for agriculture (EPPO 2019, EFSA 2022). This xylem limited plant pathogenic bacterium is transmitted by xylem-sap feeders and *Philaenus spumarius* L. (1758) (Hemiptera: Aphrophoridae) is the only epidemiologically relevant vector of *X. fastidiosa* in Europe (EFSA 2015, Cornara et al. 2019). Therefore, a detailed understanding of its ecology and phenology is essential for developing an accurate forecasting model for controlling the vector. *Philaenus spumarius* is a univoltine species, with a single generation per year, which has an ovarian parapause and a winter diapause in the egg stage (Morente et al. 2018b, Antonatos et al. 2019, Bodino et al. 2020). Eggs overwinter until hatching occurs in early spring. After egg hatching, the pre-imago (nymph) passes through five instars and nymphal development takes about 5–6 wk until they become adults (Weaver and King 1954, Whittaker 1965, Ossiannilsson 1981, Fielding et al. 1999). In the fall mature females lay masses of eggs on plant debris on the soil until they die during winter (Morente et al. 2018b, Dongiovanni et al. 2019, Antonatos et al. 2019).

The study of the developmental times of ectotherms as a function of temperature, in particular insects, has a long history (Rebaudo and Rabhi 2018). Insects need specific accumulations of heat units (HU) to reach certain development stages, which are commonly defined by the growing degree days (GDD) (also referred to as degree-days, heat units, or thermal units) (Herms 2004). Basically, the GDD is a measure of heat accumulation over time-based on insect development rates at temperatures between the lower and upper limits. Usually, the function that describes the temperature response exhibits a unimodal form, with minimum and maximum temperatures below or above which no development occurs.

Several authors have established correlations between *P. spumarius* phenology and temperature. Chmiel and Wilson (1979) estimated the upper and lower thresholds for nymphal development of *P. spumarius* in America by using the lowest coefficient of variation (CV) method described by Arnold (1959) and they calculated the HU by using the method described by Sevacherian et al. (1977). Similarly, Zajac et al. (1989) fitted linear regression functions to study the nymphal development of *P. spumarius*. One of the critical components of using GDD models is the determination of the starting points for degree-day accumulations (Kim et al. 2020). Ideally, it should be set up when insect development begins. The starting point of egg development of *P. spumarius* is assumed to happen after a winter diapause. In the GDD model introduced by Chmiel and Wilson (1979), they arbitrarily set the GDD accumulation starting point at the 1st of January. Nevertheless, the precise date when egg development starts is totally unknown.

Forty years later, Bodino et al. (2019) calculated the days of development (DD) of the nymphal stages in the Apulia and Liguria regions of Italy, as a function of the number of hours in one year that was above a minimum temperature (8°C). Minimum temperature was calculated from assays performed at fixed temperatures (unpublished data). Thereafter, Beal et al. (2021) adapted the formula from Bodino et al. (2019) to study *P. spumarius* phenology in north coastal California, obtaining similar results, although slightly prolonged development for *P. spumarius* was observed in California compared to Italy. Despite both studies shed light for better understanding the phenology of nymphal development in relation with temperature, a forecasting tool to predict egg hatching and determine the precise moment for applying control measurements is still lacking. Furthermore, in many studies, a simple linear approximation was used to compute the GDD metric and used only the minimum temperature threshold (Johnson et al. 1998, Campbell and Hanula 2007, Bodino et al. 2019, Beal et al. 2021). Alternatively, complex nonlinear approximations have been used (Logan et al. 1976, Lactin et al. 1995, Briere et al. 1999). However, Quinn (2017) concluded in his extensive critical review that for most studies conducted thus far, more complex functions performed poorly relative to simpler ones. Moreover, degree-day models are usually based on laboratory assays that aim to experimentally determine the minimum, optimum, and maximum development temperatures, and such assays typically involve fixed temperature-controlled experiments (e.g., Diaz et al. 2007).

In the present study, we developed a degree-day model to forecast the egg hatching of *P. spumarius* by following the logical steps for model construction (Overton 1977): i) Data collection: We obtained field data from independent experiments conducted on egg hatching of *P. spumarius* in their natural environments and on-site temperature recordings at specific locations. ii) Model construction: Our model was based on a multilinear temperature response function with the minimum, optimal and maximum temperatures. iii) Model calibration: We calibrated the model and obtained the minimum, optimal and maximum temperatures that control egg development by using an optimization procedure to determine the parameters that provided the best fit to the available experimental data. iv) Model validation: Our model was validated with systematic and independent survey data of newborn nymphs in different regions in Spain. v) Model extension: The model was extended to predict egg hatching throughout the entire territory. Moreover, our GDD model was used as a decision support tool to determine the best timing for applying control measures to manage vector populations.

## Materials and Methods

### Insects and Plants

**Fig. 1.**
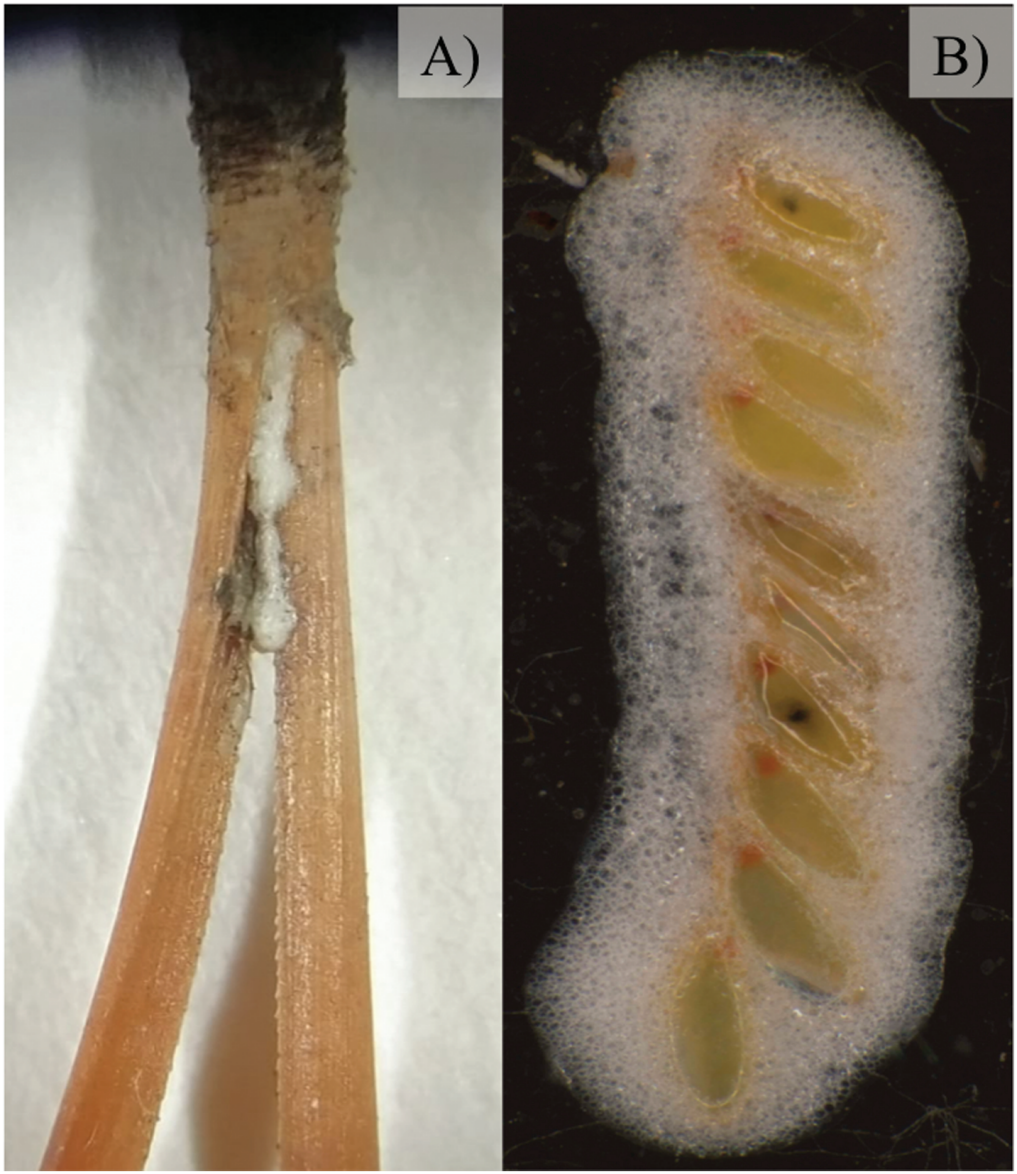
A) *Philaenus spumarius* newly laid egg masses on a dry pine needle. B) *Philaenus spumarius* egg mass.

A total of 100 adult individuals of *P. spumarius* were collected at ‘Pinilla del Valle’ (Madrid, Centre Spain) (coordinates: 40.611108, −4.263008) during spring-summer, 2020, and were maintained inside bug-dorm cages (0.5 × 0.5 × 0.5 m) on *Sonchus oleraceus* L. plants (4–5 leaf stage) in a net house with no environmental controls (Temperature °C: MEAN + SE = 16 + 0.3, Max: 31.7, Min: 7.4; Humidity RH%: MEAN + SE = 63.5 + 1.3, Max: 99.6; Min: 20.0) at the ICA-CSIC facilities (Madrid, Spain). The proportion of sexes was one female per two males to ensure mating (Morente et al. 2018a, 2021). To obtain egg masses for the assays, which were conducted in October 2020, dry pine needles were placed on the substrate below the *S. oleraceus* plants to facilitate oviposition (Morente et al. 2018a). The pine needles were checked once per week from 6-X-2020 to 4-XI-2020 to identify the egg masses (Fig. 1A, B). The dates when egg masses were observed on the pine needles were recorded. A total of 262 egg masses from five different oviposition dates were used in the field assay. The oviposition periods were the days preceding i) 8-X-2020, ii) 14-X-2020, iii) 22-X-2020, iv) 29-X-2020, and v) 4-XI-2020.

### Data Collection: Monitoring Egg Hatching of *P. spumarius* Under Field Conditions

The egg masses from each oviposition date were divided into four equal parts, and each part was transferred to a different field location on the day after the eggs were collected. The four locations used for our field study consisted of natural environments located in central Spain. These sites were selected at different altitudes to provide a gradient in climate conditions: i) Alcalá de Henares (588 m) (coordinates: 40.521133, −3.290865); ii) Bustarviejo (1,222 m) (coordinates: 40.691827, −3.767162); iii) Mataelpino (1,086 m) (coordinates: 40.736439, −3.944309); and iv) Pedrezuela (880 m) (coordinates: 40.761003, −3.619030). At each field location, the egg masses were kept on an open 5.5-cm-diameter petri dish with small holes below to facilitate drainage. The petri dishes with egg masses were placed inside floorless mesh cages (50 cm height and 45 cm diameter) (Fyllen cloth basket 79 l, Inter IKEA Systems, Sweden). Two cages were placed at each field location: The cages were divided into four equal sectors by using cellulose acetate sheets. Since we had five oviposition dates, two and three plants were placed inside each of the two cages, respectively (Supp. Fig. S1). Egg masses from a given oviposition date were placed in a single plant and sector of the cage to avoid mixing nymphs after emergence. A few weeks before the first nymphs were expected to emerge in the field (e.g., 25-II-2021), one *S. oleraceus* plant was transplanted inside each cage division where the egg masses were placed to feed the newborn nymphs after emergence (Supp. Fig. S1). After transplanting, the egg masses were transferred from the petri dishes to the bare soil below the plants. In Mataelpino, the potted *S. oleraceus* plants inside the cages were watered weekly as opposed to the rainfed field locations. At this time, we checked each plant once a week to record egg hatching when newborn nymphs were observed. The newborn emerged nymphs were removed from the plants to facilitate further observations. All plants were inspected until no newborn nymphs were observed for two consecutive weeks.

Temperature and relative humidity (RH) were monitored hourly at each field location with temperature and humidity data loggers (OM-EL-USB-2, Omega Engineering, INC, Norwalk, Connecticut, USA) during the entire duration of the experiments. One data logger not exposed to direct sunlight was placed inside each cage at each field location to obtain the same temperature and RH data as that experienced by the egg masses. The data collected from the data loggers were downloaded every two weeks until the emergence of newborn nymphs. Thereafter, data were downloaded once a week. In the few cases where the data loggers failed to record weather data due to extreme weather conditions, the missing data were supplemented with external data collected from the nearest official meteorological station (Agencia Estatal de Meteorología de España, AEMET, Spain). After monitoring egg-hatching in each of our four experimental sites, we computed RH as mean values experienced by each egg during the experimental period per field site. Furthermore, we calculated the Wasserstein distance to measure the distance between probability distributions of GDD accumulation between each pair of field sites (two by two) (Panaretos and Zemel 2019).

### Model Construction: GDD-based Model

In the current study, we used a multilinear temperature response function with minimum or base (*T*_*base*_), optimal (*T*_*opt*_), and maximum (*T*_*max*_) temperatures to calculate the hourly contributions to the GDD. The use of a multilinear function is based on the principles of biochemical kinetics (i.e., on Arrhenius’ Law) (Eq. 1) and is explained in detail in Supp. Document S2.


$$\begin{array}{l} f\left(T\right)=\left\{ \begin{array}{l} 0ifT<Tbase\\ T-TbaseifTbase\leq T<Topt\\ m\cdot T+nifTopt\leq T<Tmax\\ 0ifT\geq Tmax \end{array}\right.withm=\frac{-Topt-Tbase}{Tmax-Topt},n=-m\cdot Tmax \end{array}$$ (1)

In our model, the accumulated GDD values are directly related to the cumulative probabilities of egg hatching using the cumulative density function of the Weibull distribution where *k* > 0 and λ > 0 are the shape and scale parameters of the Weibull distribution, respectively.


$$\begin{array}{l} Phatching\left(GDD\right)=1-e^{\left(-{\left(GDD/\lambda \right)}^{k}\right)} \end{array}$$ (2)

The starting date for GDD accumulation was the date when the winter diapause of *P. spumarius* ended. This date, in principle, should be determined by some metric of accumulation of cold temperatures (below a given threshold). However, the precise hours or days needed under a given temperature -cold requirement- and the precise length of the winter diapause of *P. spumarius* are unknown. Thus, we compared different arbitrarily fixed dates of diapause ending (January 1st, December 1st, and November 1st), and later validated the model with data obtained from field observations of newborn nymphs in the field from systematic surveys at different sites in Spain over the last six years (Supp. Table S3). The modeling was performed in Python programming language (Van Rossum and Drake 1994).

### Model Calibration

The goal of our model was to provide estimates of the cumulative hatching probabilities of *P. spumarius* eggs by using the temperature data provided by the field experiments described above transformed into a GDD metric. All data were treated independently of location, and then pooled together. The experimental cumulative hatching probability at a given time was calculated as the number of nymphs that had already emerged at that given time over the total number of nymphs that had emerged by the end of the assay. Then, the cumulative GDD values between the end of diapause and the egg hatching dates had to be calculated so that the cumulative hatching probabilities could be expressed as functions of the accumulated GDD values. In this way, we could fit Eq. (2) to the experimental data and obtain an effective model for predicting egg hatching. However, the relationship between the cumulative hatching probabilities and accumulated GDD values depends on how the accumulated GDD is calculated; that is, it depends on the GDD (t) profile, namely on the cardinal temperatures, Eq. (1), and the starting date of accumulation. Thus, we first calibrated our model to obtain the GDD temperature profile and a starting date of GDD accumulation that provided the best-predicted hatching probabilities for our dataset.

We selected three plausible starting dates for GDD accumulation as described above: 1st of November, 1st of December, and 1st of January. Then we calculated the optimal GDD temperature profiles, by finding the values of *T*_*base*_, *T*_*opt*_, and *T*_*max*_ that gave the best fit of Eq. (2), which depends on Eq. (1), to the observed hatching probabilities of our dataset (the four field experimental sites pooled together). More precisely, we tested all the combinations with *T*_*base*_ from 4 to 12ºC, *T*_*opt*_ from 20 to 28ºC, and *T*_*max*_ from 30 to 42ºC in steps of 0.2ºC, predicting the hatching probabilities using Eq. (2) by using the accumulated GDDs computed with each profile and each starting date using Eq. (1). Finally, we selected the optimal profile (the optimal values of *T*_*base*_, *T*_*opt*_, and *T*_*max*_) to define the profile that minimized the error between Eq. (2) and the experimental dataset for each starting date. To fit Eq. (2) to the experimental data we used the nonlinear least squares method implemented in the ‘scipy.optimize.curve_fit’ method in Python programming language.

### Model Validation

To validate our model, we predicted the egg-hatching probabilities in the Iberian Peninsula by using the existing dataset from field observations recorded from 2016 to 2021 in Spain (Supp. Table S3) using the calibrated model considering three different dates of diapause termination date: 1st of November, 1st of December, and 1st of January. For each diapause termination date considered (1st of November, 1st of December, and 1st of January), an optimal GDD temperature profile can be calculated from our dataset (the 4 experimental sites). The hourly temperature data for these years were retrieved from the ERA5-Land dataset, which has a spatial resolution of 0.1° × 0.1º. Then, we computed the hatching probabilities with daily resolution and compared the predictions with the observational data from the field that were obtained from systematic surveys of newborn nymphs in different regions of Spain during six consecutive years (2016–2021) (Supp. Table S3). These data were obtained from systematic field surveys conducted by different researchers from public institutions in Spain (Andalucía, La Rioja, Madrid, Murcia, and Valencia) (see Acknowledgments). These data consisted of several records of GPS coordinates and dates where newborn nymphs were found in Spain, so we expected that our model would predict high probabilities of egg hatching (*>*50%) for each date and location where newborn nymphs were observed. Therefore, we conducted a validation process to find out which diapause termination date fitted best to our six-year dataset.

### Control Timing of *P. spumarius* Nymphs

After model validation, we used our calibrated and validated model to investigate the best timing to adopt control actions against *P. spumarius* nymphs based on field observations. To do so, we calculated the daily hatching probabilities of *P. spumarius* eggs in the Iberian Peninsula based on ERA5-Land temperature data (Muñoz Sabater 2019) and simulated applications of control actions at several probability levels. Thereafter, we calculated the efficacy of applying a control action at a given time, which is defined as the percentage of nymphs targeted in the field before they reach the adulthood if a control action was taken at that given time. Thus, the following algorithm was developed to determine the timing of control actions based on our model predictions. First, we selected a plausible range of diapause termination dates as the starting dates for our simulations. Although we calibrated and validated our model for a particular diapause termination date, we investigated if different dates with the same temperature profile were able to provide better results; then, we selected the ending date of each simulation as a plausible date on which no more egg hatching was expected. Afterward, we selected several probability levels to apply control actions (i.e., when any of these selected levels is crossed in any location, we simulated the application of a control action). For each diapause termination date in each simulation, the daily hatching probabilities were computed until the end of the simulation using the hourly temperature data from ERA5-Land. Then, we saved the dates at which every selected probability level (control action thresholds) was crossed at each location in which we have data for the presence of newborn nymphs in the field. (Supp. Table S3). Finally, the lags between the control action dates given by each probability level and observation dates were computed.

Each selected hatching probability (which matches an action date) has a given control action efficacy. It is known that nymphal development lasts for approximately 5–6 wk until they become adults, but the developmental rates decrease when temperatures are low and could extend to at least 100 days (Weaver and King 1954, Yurtsever 2000, Bodino et al. 2019). Thus, we followed a conservative approach to compute the control action efficacies and defined successful nymph control only if the action was applied after the newborn nymphs were observed and there was a lag of less than 30 days.

According to our results, there is high variability in the hatching dates (see the results section). Thus, to overcome this intrinsic variability in the timing of egg hatching of *P. spumarius,* we developed a strategy based on deploying control actions at two different dates to target the maximum number of nymphs as completely as possible. A list of combinations of two probability levels was selected (e.g., the first at 50% and the second at 90%) to apply control actions on two different dates. Moreover, the efficacies of applying control actions at a single date or at two different dates were compared.

In addition, we developed an R package script to calculate the daily cumulative hatching probabilities of *P. spumarius* eggs based on suitable temperature data. This package also calculates the optimum time to apply the first and second control actions, depending on the hatching probability achieved. This package and all relevant information regarding its use can be found on the GitHub repository: https://github.com/agimenezromero/PSEggHatching.

## Results

### Nymphal Emergence

**Fig. 2.**
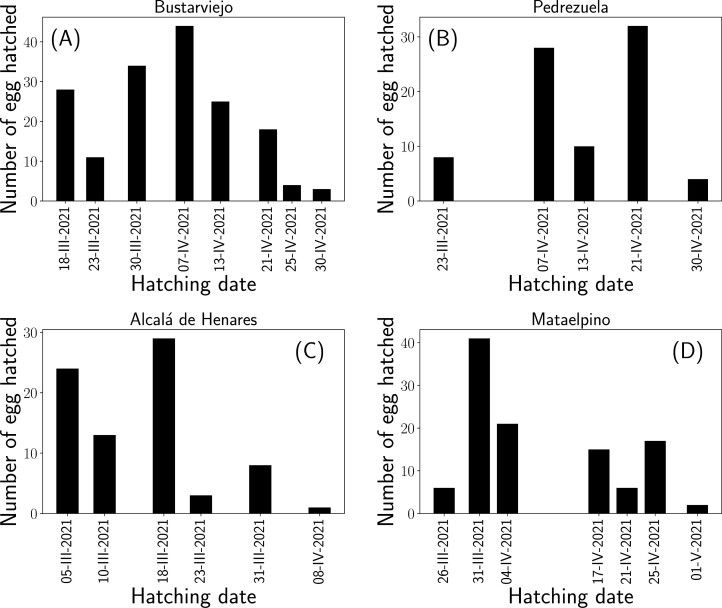
Number of eggs hatched per date at each field site. The five oviposition dates were merged per each hatching date. Scouting weeks: 1 = 22-II-2021 to 28-II-2021; 2 = 01-III-2021 to 07-III-2021; 3 = 08-III-2021 to 14-III-2021; 4 = 15-III-2021 to 21-III-2021; 5 = 22-III-2021 to 28-III-2021; 6 = 29-IV-2021 to 04-IV-2021; 7 = 05-IV-2021 to 11-IV-2021; 8 = 12-IV-2021 to 18-IV-2021; 9 =19-IV-2021 to 25-IV-2021; 26-IV-2021 to 02-V-2021. A) Bustarviejo. B) Pedrezuela. C) Alcalá Henares. D) Mataelpino.

A total of 435 *P. spumarius* nymphs emerged in the field assays at the four experimental sites. Newborn nymphs were detected feeding on *S. oleraceus* plants from 5-III-2021 to 1-V-2021. The first detection of newborn nymphs was recorded on 5-III-2021 at Alcalá de Henares (588 m), on 18-III-2021 at Bustarviejo (1,222 m), on 23-III-2021 at Pedrezuela (880 m) and 26-III-2021 at Mataelpino (1,086 m). Nymph emergences occurred at different moments and extended for approximately two months at all of the studied sites (Fig. 2). The results for the nymphal emergence dates per field location show that the intrinsic randomness of the hatching process is quite broad, which exhibits variations of up to one month for eggs subjected to the same environmental conditions. No correlation between the oviposition date and hatching date was observed after pooling data from all of the field sites (ρ = −0.036). Furthermore, there was no or poor correlation between the oviposition date and hatching date within specific field sites: Alcalá Henares (ρ = −0.192), Bustarviejo (ρ = −0.289), Mataelpino (ρ = −0.016), and Pedrezuela (ρ = −0.316).

### Model Calibration

We calibrated the GDD temperature profile assuming different diapause termination dates: 1st of November, 1st of December, and 1st of January. We obtained an unrealistic temperature profile with high *T*_*opt*_ and *T*_*max*_ values when starting the accumulation of GDD on the 1st of January (*T*_*base*_ = 9.2°C, *T*_*opt*_ = 27.6°C, and *T*_*max*_ = 41.8°C) and 1st of November (*T*_*base*_ = 9°C, *T*_*opt*_ = 27.4°C, and *T*_*max*_ = 41.2°C). In contrast, more appropriate and realistic results were obtained when selecting the 1st of December as the diapause termination date (*T*_*base*_ = 9.2°C, *T*_*opt*_ = 23.4°C, and *T*_*max*_ = 34.2°C).

**Fig. 3.**
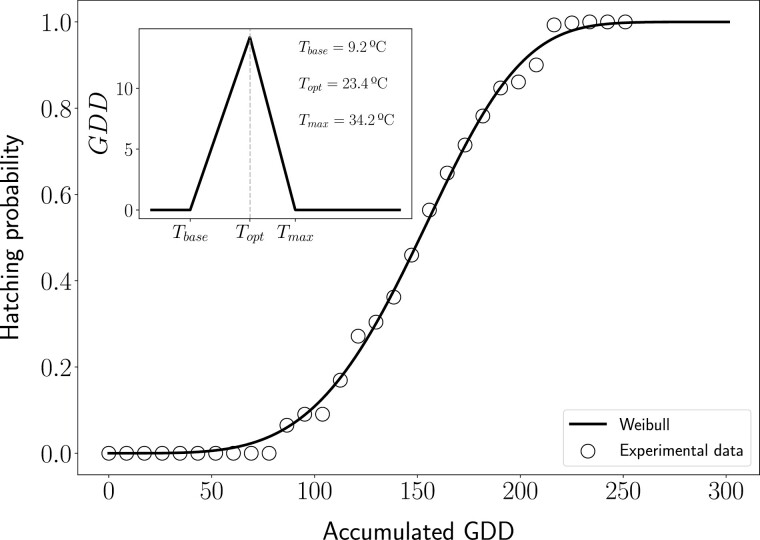
Model calibration considering diapause breakage on December 1st. The main figure shows the hatching probability as a function of the accumulated GDD value. Dots represent the experimental data, and the black solid line shows the best fit using Eq. (1). The inset shows the GDD profile that is used to calculate the accumulated GDD values, which yielded the best fit between Eq. (1) and experimental data.

As a visual example, Fig. 3 shows the final result of the model after the calibration procedure for the diapause termination date for 1st December (model calibration for diapause termination date for 1st of January and 1st of November are shown in Supp. Document S4). The dots represent the cumulative hatching probabilities that were retrieved from the dataset as a function of the accumulated GDD values. This accumulated GDD value was obtained using the optimal GDD profile that is shown in the inset (*T*_*base*_ = 9.2°C, *T*_*opt*_ = 23.4°C, and *T*_*max*_ = 34.2°C). The black line is the best fit of Eq. (1) to the experimental data (dots) that was obtained with *k* = 4.34 and λ = 164.86, which had a relative error of 1%.

### Model Validation and Predictions of Egg Hatching Dates in the Iberian Peninsula

It was found that our model predictions were only consistent with the field data when the diapause termination date was set to the 1st of December (Supp. Video S5 https://zenodo.org/record/7465753). When assuming the 1st of January, the model predicts egg hatching too late so that nymphs are observed about a month before our model predicts high hatching probabilities (Supp. Video S5 https://zenodo.org/record/7465753). On the other hand, when assuming November 1st, the model predicts a high probability of egg hatching too early, about 2 months before field observations (Supp. Video S5 https://zenodo.org/record/7465753). Nevertheless, considering the diapause termination as the 1st of December, some differences were also observed between model predictions and field observations from systematic surveys (Supp. Table S3) in southern Spain (latitudes below 40º) and northern Spain (latitudes above 40º). As shown in Supp. Video S5, in the South, 1st instar nymphs were detected in the field, when the model predicted high hatching probabilities (above 80%), while in the North 1st instar nymphs are detected when the model predicted low hatching probabilities (20–30%).

### Impact of RH on GDD for Hatching and GDD Accumulation in the Field Sites

**Fig. 4.**
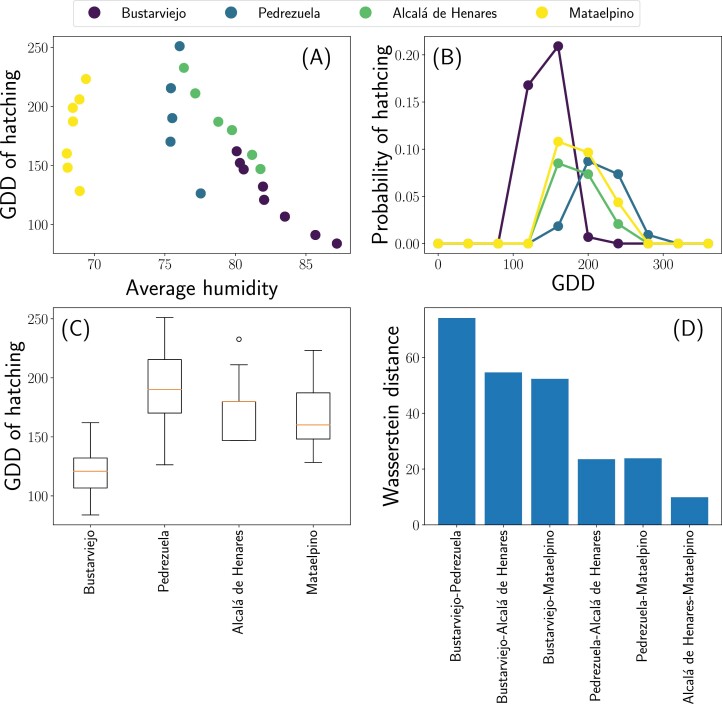
A) GDD accumulation metric at the moment of egg hatching versus the RH values at the experimental locations. Each data point corresponds to one hatching event. B) Probability density function of egg hatching as a function of accumulated GDD C) Distribution of GDD needed for egg hatching on each field site. The horizontal orange lines represent median values. Boxes extend from the 25th to the 75th percentile of each group’s distribution of values, and vertical extending lines indicate the range of values. D) Wasserstein Distance (WD) between probability distributions of GDD accumulation between each pair of field sites (two by two).

After model calibration and validation, considering diapause breakage on December 1st we explored the impact of RH on GDD needed for hatching. As shown in Fig. 4A, we didn’t record humidity values below 60% in Mataelpino and not below 75% in the rest of field points therefore, the relationship between RH and GDD at RHs below these values is unknown. Furthermore, there is no clear pattern or relation between RH and GDD in any of the field sites (Fig. 4A). As shown in Fig. 4B, the probability density function of egg hatching as a function of accumulated GDD is very similar in all field sites, except Bustarviejo and the boxplot of Fig. 4C again shows that the GDD needed for hatching is similar in all locations except Bustarviejo. More quantitatively, Fig. 4D shows the Wasserstein distance among the distributions, showing that Bustarviejo is the only one different from the others. Temperatures and RH registered in the four field locations are shown in Supp. Document S6.

### Decision-Support Tool to Determine the Best Timing for controlling *P. spumarius* Nymphs

**Fig. 5.**
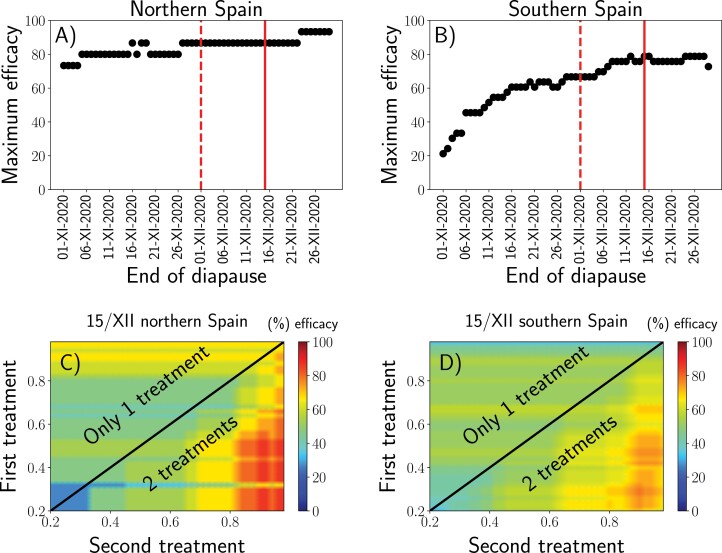
(A and B) Maximum control action efficacies for each diapause breakage date from the selected range of dates to develop the algorithm (from November 1st to December 30th). The dashed line shows the diapause termination selected for model development (December 1st) and the solid line indicates the diapause termination date to achieve the maximum efficacy (December 10th in the north and December 12th in the south). A) Efficacy in northern Spain (latitude >40°N). B) Efficacies in southern Spain (latitude <40°N). (C and D) Examples of the efficacies of applying a control action depending on the egg hatching probability after applying one or two treatments (C) in northern Spain when considering diapause termination on December 10th and (D) in southern Spain when considering diapause termination on December 15th. This analysis was repeated for all diapause termination dates to obtain the maximum efficacies shown in (A and B).

After the model validation, we calculated the precise timing for controlling *P. spumarius* nymphs most efficiently in different regions in Spain. The following initial conditions were fixed according to our results: i) We selected a plausible range of diapause termination dates: we selected the range 1st of November to 1st of January ii). The end date of each simulation was based on the field observations from the systematic surveys (2016–2021) (Supp. Table S3). Because the latest newborn nymphs were found on 27-V-2021 we assumed that the end date of each simulation was June 1st. With this algorithm in mind, the optimal dates for applying control actions (control timing) were selected to maximize the defined efficacy, which is the maximum percentage of targeted nymphs. In addition, we considered all possible date combinations for taking certain control actions and compared these simulations with all the hatching observations from both our assays and the systematic surveys (Supp. Table S3). This maximum efficacy changed depending on the diapause termination date (Fig. 5A–B). Consistent with the previous validation, it was found that the model efficacy is maximized if diapause termination is considered to occur in December. Interestingly, we found that assuming diapause termination in mid-December instead of 1st December is a more robust choice. In this way, if the diapause termination date slightly varies for a given particular year, we expect to maintain high efficacy (Fig. 5A–B). Furthermore, to optimize the control actions and to target the maximum number of nymphs as effectively as possible, a control strategy (control timing) applied at a single date was compared to a two-date application strategy. Our results clearly show that the best strategy consists of applying control actions at two different times to target the maximum nymphal population, achieving efficacies of 87 and 78% in northern and southern Spain, respectively (Fig. 5C–D, Supp. Tables S7–S8). In contrast, the maximum efficacy achieved by applying only one control action are 67% in northern Spain and 58% in southern Spain. Thus, by applying two control actions the targeted population increases in a 30% According to our results, for northern Spain (latitudes above 40°N) the first control actions should be taken when the accumulated egg-hatching probabilities reach 40% and the second when they reach 90%. On the other hand, for southern Spain (latitudes below 40°N), the first control actions should be taken when the accumulated egg-hatching probability reaches 30% and the second when it reaches 90% (Fig. 5, Supp. Tables S6–S7).

## Discussion

In the present work, we built a GDD model obtaining the temperature thresholds (*T*_*base*_, *T*_*max*_, and *T*_*opt*_) directly from experimental data obtained from field experiments under natural conditions. Our approach allows us to indirectly account for the effect of temperature fluctuations on the developmental rate, which is automatically incorporated into the model within the best-fit parameters. Temperature has significant nonlinear effects on insect development, which is slow when the temperatures approach either the upper or lower developmental thresholds (Murray 2020). For this reason, we provided a mathematical approach based on temperature effects on the metabolic rates of ectotherms (Gillooly et al. 2001) to model the nonlinear development rates with a multilinear function. The model calibration and validation further support our approach of using a multilinear function to calculate the GDD metric and validate the direct use of field data to accurately determine the temperature thresholds.

Chmiel and Wilson (1979) modeled nymphal development of *P. spumarius* by using temperature data from meteorological stations, while in the present work, we installed data loggers inside the cages where the eggs were developing, similarly to the model of nymphal development constructed by Bodino et al. (2019). In this way, we obtained similar data as those experienced by the eggs (Bonhomme 2000). For model construction, it is essential to understand that *P. spumarius* overwinters at the egg stage experimenting a winter diapause (Witsack 1973). Nevertheless, the precise time when egg development starts is unknown, thus establish a starting point for GDD accumulation is challenging. In the GDD model introduced by (Chmiel and Wilson 1979), they arbitrarily set the GDD accumulation starting point on the 1st of January. For our model, we tested 1st of November, 1st of December, and 1st of January as possible ending diapause dates. When we tested December 1st as the starting date for GDD accumulation the temperature profile (*T*_*min*_ = 9.2ºC, *T*_*opt*_ = 23.4, *T*_*max*_ = 34.2) was more realistic than on 1st of January (*T*_*min*_ = 9.2, *T*_*opt*_ = 27.6, *T*_*max*_ = 41.8ºC) or November 1st (*T*_*base*_ = 9°C, *T*_*opt*_ = 27.4°C, and *T*_*max*_ = 41.2°C) since maximum temperature yielded were well above the biological limit for survivorship of the insect, according to what we know about the life cycle parameters and thermal requirements of *P. spumarius* (Weaver and King 1954, Halkka et al. 2006). Furthermore, when we tested December 1st as the starting date for GDD accumulation the temperature profile (*T*_*min*_ = 9.2ºC, *T*_*opt*_ = 23.4, *T*_*max*_ = 34.2) was more realistic and the model predictions matched well the predictions of 1st instar nymph emergence in Spain giving much more convincing results (see Supp. Video S5 https://zenodo.org/record/7465753).

The main reason for the discrepancy between the dates when the diapause ends in the study conducted by Chmiel and Wilson (1979) and the earlier date that we assumed in our study is presumably related to the striking differences in the winter climate between the two regions. While the former study was conducted in West Lafayette, Indiana, USA where winter temperatures are extremely low, our study was conducted in the Iberian Peninsula where temperatures are milder. Thus, *P. spumarius* populations from different regions may have different temperature developmental thresholds or different diapause termination requirements. The induction and termination of winter diapause are crucial components of the life cycle of insect species subjected to diapause (Posledovich et al. 2015, Tougeron 2019) and a geographic variation in diapause duration depending on temperature has been previously reported by several authors (e.g. Schmidt et al. 2005, Chuche and Thiéry 2009, Salman et al. 2019). Therefore, we assumed that *P. spumarius* populations that occur in Spain are adapted to milder winters and thus, they require fewer hours of cold for diapause termination.

Before applying any control actions, the best time of application, control timing, should be defined. For controlling *P. spumarius* population is essential to apply actions at nymphal stage, before they reach adulthood, since nymphs have limited mobility while adults actively displace (Lago et al. 2021a, 2021b) and only adults contribute to the spread of *X. fastidiosa* to woody hosts (Cornara et al. 2016, 2018). There is a broad time window for nymphal emergence, about two months according to our results, and similar to those previously reported by several authors (Morente et al. 2018a, Bodino et al. 2019). In addition, nymphal development takes 5–6 wk until reaches adulthood (Weaver and King 1954, Yurtsever 2000, Bodino et al. 2019). Therefore, controlling nymphs at two different dates would target the highest percentages of nymphal populations present in the field, before they reach the adulthood. Moreover, the first control actions in the north should be taken when the accumulated egg-hatching probability reaches 40% and the second when it reaches 90%. For southern Spain, the first control actions should be taken when the accumulated egg-hatching probability reaches 30% and the second when it reaches 90%.

In the present study, we provided an R package script for practical use to compute GDDs in each location based on the local temperatures, and in turn, the model can determine the hatching probability at a given site and date (https://github.com/agimenezromero/PSEggHatching). Given an input dataset of hourly temperatures in a precise location from the starting date of GDD accumulation to a given date where the model is run, the package provides the current probability level for egg hatching at that given date and precise location. So, further explained, first, the user should somehow obtain hourly temperature data starting on the 1st of December. Then, each day after the 1st of December, the user could introduce this CSV (comma-separated values) data file containing up-to-date hourly temperature into the R program and the output would be the probability level that has been achieved up to that given date. If for that current date the probability reaches the first or second threshold the program warns the user that a treatment should be performed.

The developed model could be used as a decision-making tool to make recommendations regarding the best timing to adopt certain control actions against the main vector of *X. fastidiosa* and to reduce the risk of disease spread. Furthermore, these fitted models can provide estimates of *P. spumarius* biology, and potentially give valuable knowledge in future studies to predict biological aspects of the life cycle of other insect pests.

## Supplementary Material

nvad013_suppl_Supplementary_Figure_S1

nvad013_suppl_Supplementary_Document_S2

nvad013_suppl_Supplementary_Table_S3

nvad013_suppl_Supplementary_Document_S4

nvad013_suppl_Supplementary_Document_S6

nvad013_suppl_Supplementary_Tables_S7_S8
