# Supplementary figures and images for: Degree-day-based model to predict egg hatching of Philaenus spumarius (Hemiptera: Aphrophoridae), the main vector of Xylella fastidiosa in Europe
Source: Environ Entomol. 2023 Apr 19;52(3):350–9. doi: 10.1093/ee/nvad013 (PMC10272708; doi:10.1093/ee/nvad013)

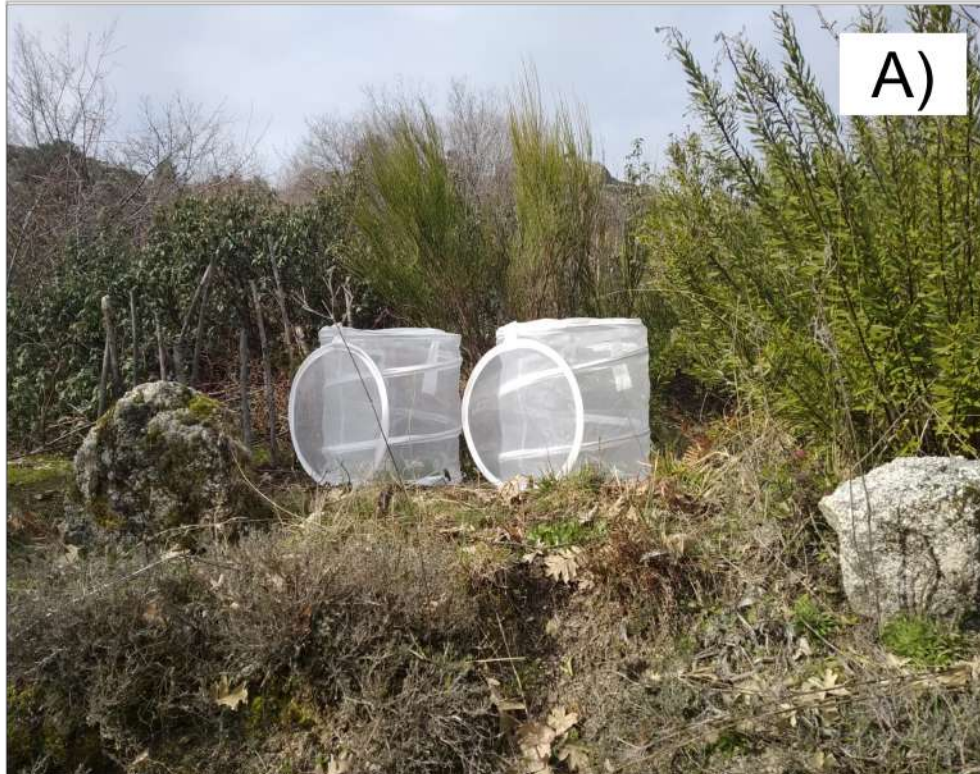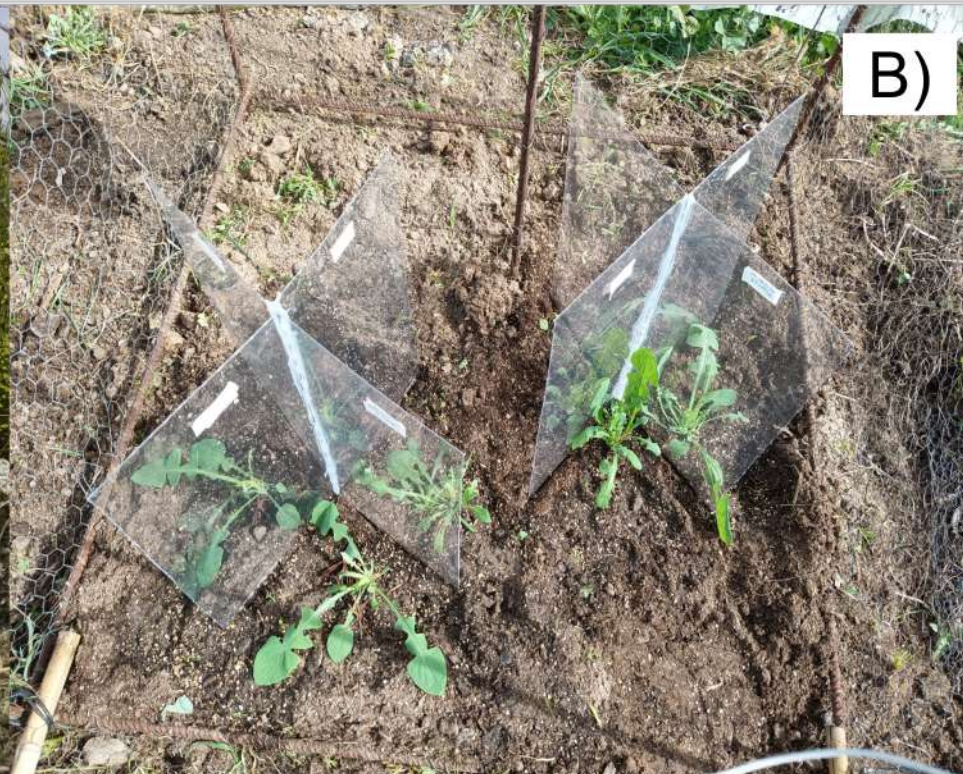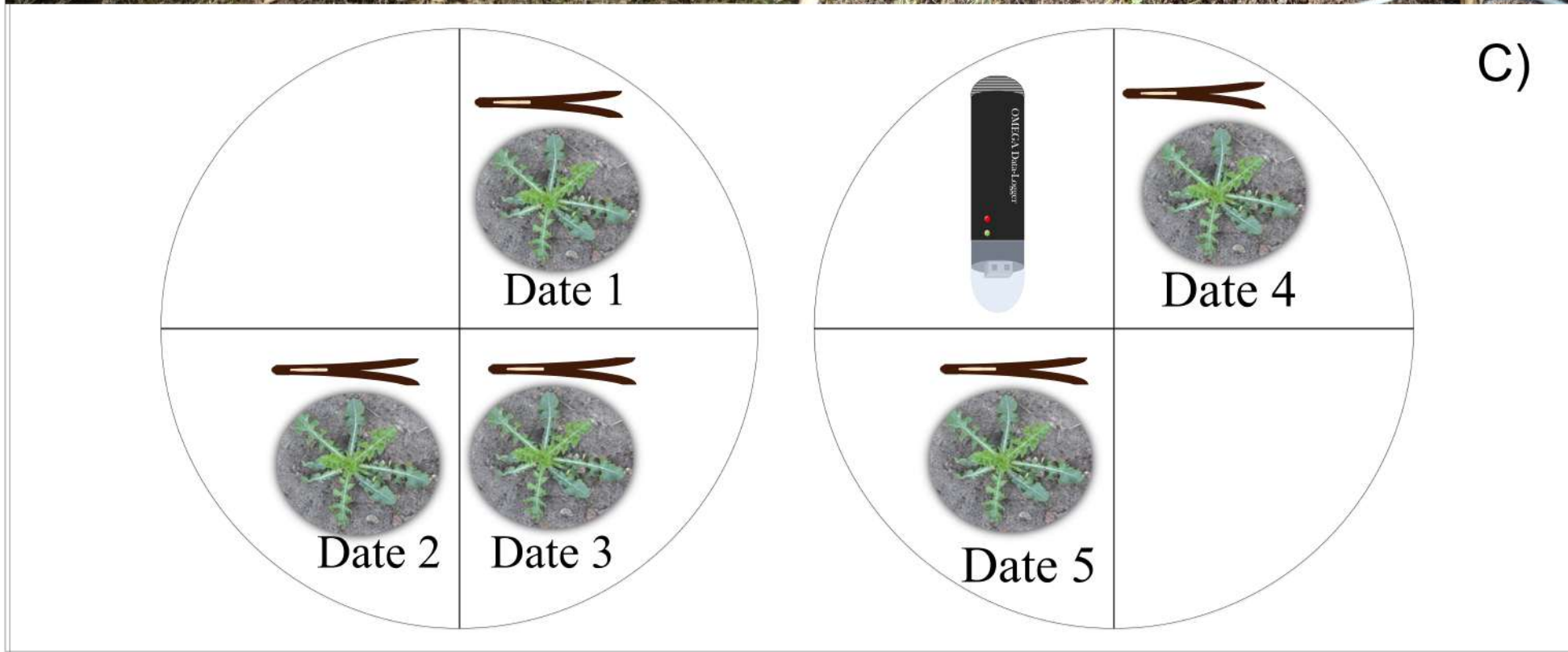

Supplement: nvad013_suppl_Supplementary_Figure_S1 [file nvad013_suppl_supplementary_figure_s1.pdf]
