# Supplementary material for: Degree-day-based model to predict egg hatching of Philaenus spumarius (Hemiptera: Aphrophoridae), the main vector of Xylella fastidiosa in Europe
Source: Environ Entomol. 2023 Apr 19;52(3):350–9. doi: 10.1093/ee/nvad013 (PMC10272708; doi:10.1093/ee/nvad013)
Supplement: nvad013_suppl_Supplementary_Table_S3 [file nvad013_suppl_supplementary_table_s3.pdf]

Detection Date	Field location	Region	Latitude	Longitude
17-04-18	Arnedo	La Rioja	42.0012839	-1.99117916
20-04-18	Rodezno	La Rioja	42.5291667	-2.82861
27-01-20	Cartagena	Murcia	37.611866	-0.750929
11-02-20	El Berro	Murcia	37.887945	-1.493203
18-02-20	Jumilla	Murcia	38.492305	-1.189841
11-02-20	Valle Perdido	Murcia	37.927495	-1.148376
18-02-20	Yecla	Murcia	38.618762	-1.127371
29-01-21	Cartagena	Murcia	37.611866	-0.750929
06-02-21	Jumilla	Murcia	38.442847	-1.316243
05-02-21	Jumilla	Murcia	38.492305	-1.189841
29-01-21	Valle Perdido	Murcia	37.927495	-1.148376
03-02-16	Puerto Moral	Huelva	37.87485635	-6.464657663
11-02-16	Castillo de las Guardas	Sevilla	37.68167052	-6.252431569
29-03-16	Morón	Sevilla	37.10039243	-5.377321998
15-02-17	Castillo de las Guardas	Sevilla	37.6688293	-6.251865571
14-03-17	Constantina	Sevilla	37.862028	-5.57736391
07-03-17	El Ronquillo	Sevilla	37.65966196	-6.156940406
21-04-20	Colmenar Viejo	Madrid	40.691917	-3.767194
29-04-20	Colmenar Viejo	Madrid	40.691917	-3.767194
20-04-20	IMIDRA	Madrid	40.521133	-3.290865
16-04-20	ICA	Madrid	40.43961581	-3.687287572
09-02-22	IMIDRA Alcalá Henares	Madrid	40.521133	-3.290865
20-04-18	Alberite	La Rioja	42.4183333	-2.44083
19-04-18	Morata Tajuña	Madrid	40.231134	-3.450696
13-03-18	Constantina	Sevilla	37.861944	-5.576111
11-02-20	Alhama de Murcia	Murcia	37.838723	-1.467839
18-02-20	Jumilla	Murcia	38.442847	-1.316243
19-02-21	Alhama de Murcia	Murcia	37.838723	-1.467839
19-02-21	El Berro	Murcia	37.887945	-1.493203
17-02-21	Yecla	Murcia	38.618762	-1.127371
17-02-21	Yecla	Murcia	38.7307	-1.13601
10-03-16	Constantina	Sevilla	37.86131266	-5.57711167
18-02-16	Osuna	Sevilla	37.15849409	-5.139528214
09-03-17	Algodonales	Cádiz	36.88516931	-5.451834299
13-03-18	Constantina	Sevilla	37.862028	-5.57736391
14-03-17	Osuna	Sevilla	37.15982699	-5.139081596
26-02-18	Castillo de las Guardas	Sevilla	37.66339545	-6.247655085
16-04-20	Bustarviejo	Madrid	40.691827	-3.767162
16-04-20	Colmenar Viejo	Madrid	40.691917	-3.767194
18-03-18	Guadalest	Alicante	38.662778	-0,00278
07-04-18	Osuna	Sevilla	37.1594444	-5.1394
27-05-21	Santa María de la Alameda	Madrid	40.611108	-4.263008
24-03-21	Jumilla	Murcia	38.4447945	-1.3157153
24-03-21	Yecla	Murcia	38.577469	-1.1976939
16-04-20	Bustarviejo	Madrid	40.691827	-3.767162
13-05-20	Bustarviejo	Madrid	40.691827	-3.767162
17-04-20	IMIDRA	Madrid	40.521133	-3.290865
13-04-21	Sierra Aracena	Huelva	37.861016	-6.484765
29-04-21	Villanueva Cañada	Madrid	40.454418	-4.005871
