# Supplementary material for: Degree-day-based model to predict egg hatching of Philaenus spumarius (Hemiptera: Aphrophoridae), the main vector of Xylella fastidiosa in Europe
Source: Environ Entomol. 2023 Apr 19;52(3):350–9. doi: 10.1093/ee/nvad013 (PMC10272708; doi:10.1093/ee/nvad013)

Model Calibration considering diapause breakage on January 1st

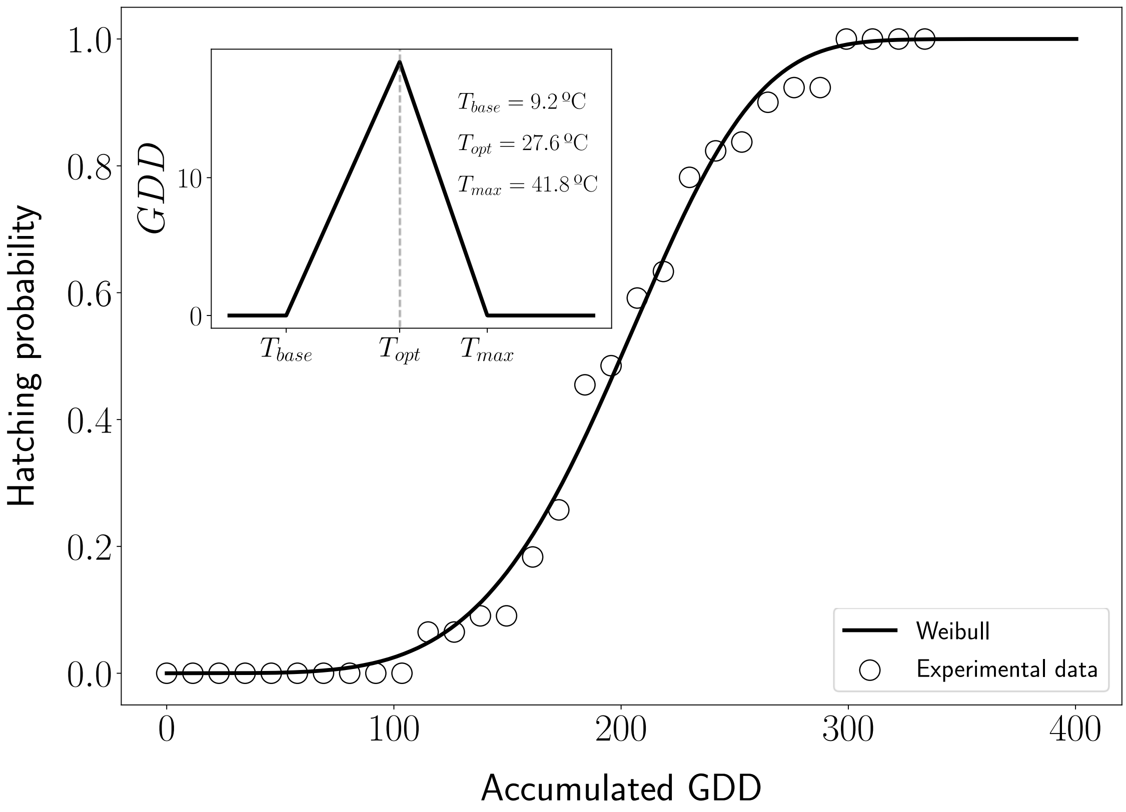

Model Calibration considering diapause breakage on November 1st

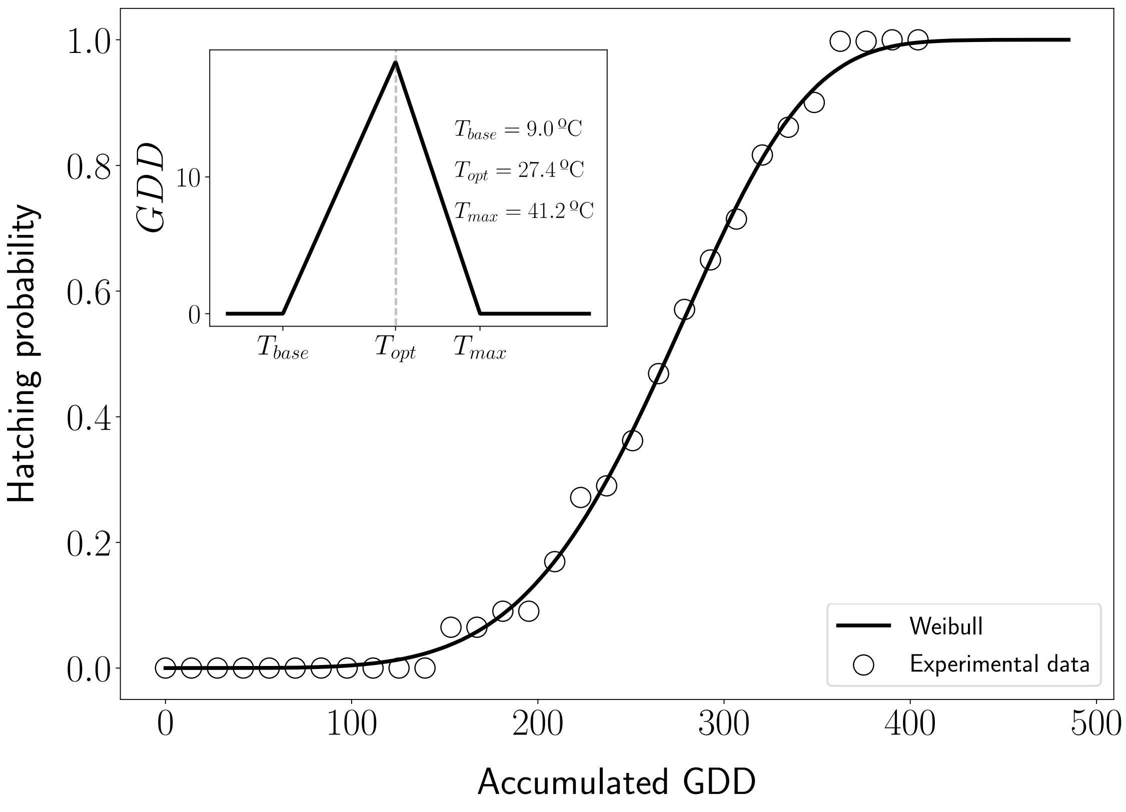

Supplement: nvad013_suppl_Supplementary_Document_S4 [file nvad013_suppl_supplementary_document_s4.pdf]
