# Supplementary material for: Degree-day-based model to predict egg hatching of Philaenus spumarius (Hemiptera: Aphrophoridae), the main vector of Xylella fastidiosa in Europe
Source: Environ Entomol. 2023 Apr 19;52(3):350–9. doi: 10.1093/ee/nvad013 (PMC10272708; doi:10.1093/ee/nvad013)
Supplement: nvad013_suppl_Supplementary_Document_S6 [file nvad013_suppl_supplementary_document_s6.pdf]

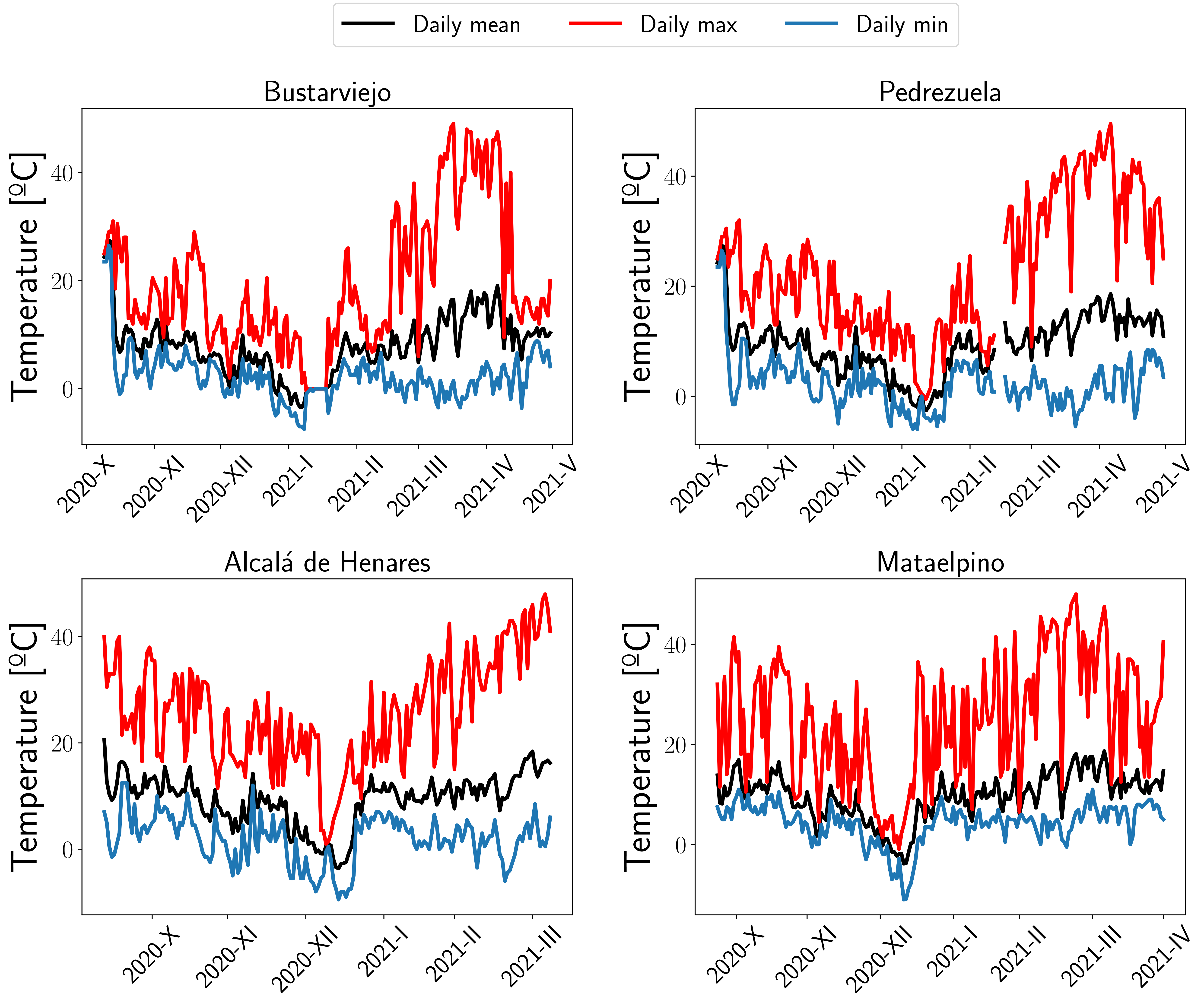
Daily mean
Daily max
Daily min
Bustarviejo
Pedrezuela
Alcalá de Henares
Mataelpino
Temperature [ºC]
0
20
40
2020-X
2020-XI
2020-XII
2021-I
2021-II
2021-III
2021-IV
2021-V

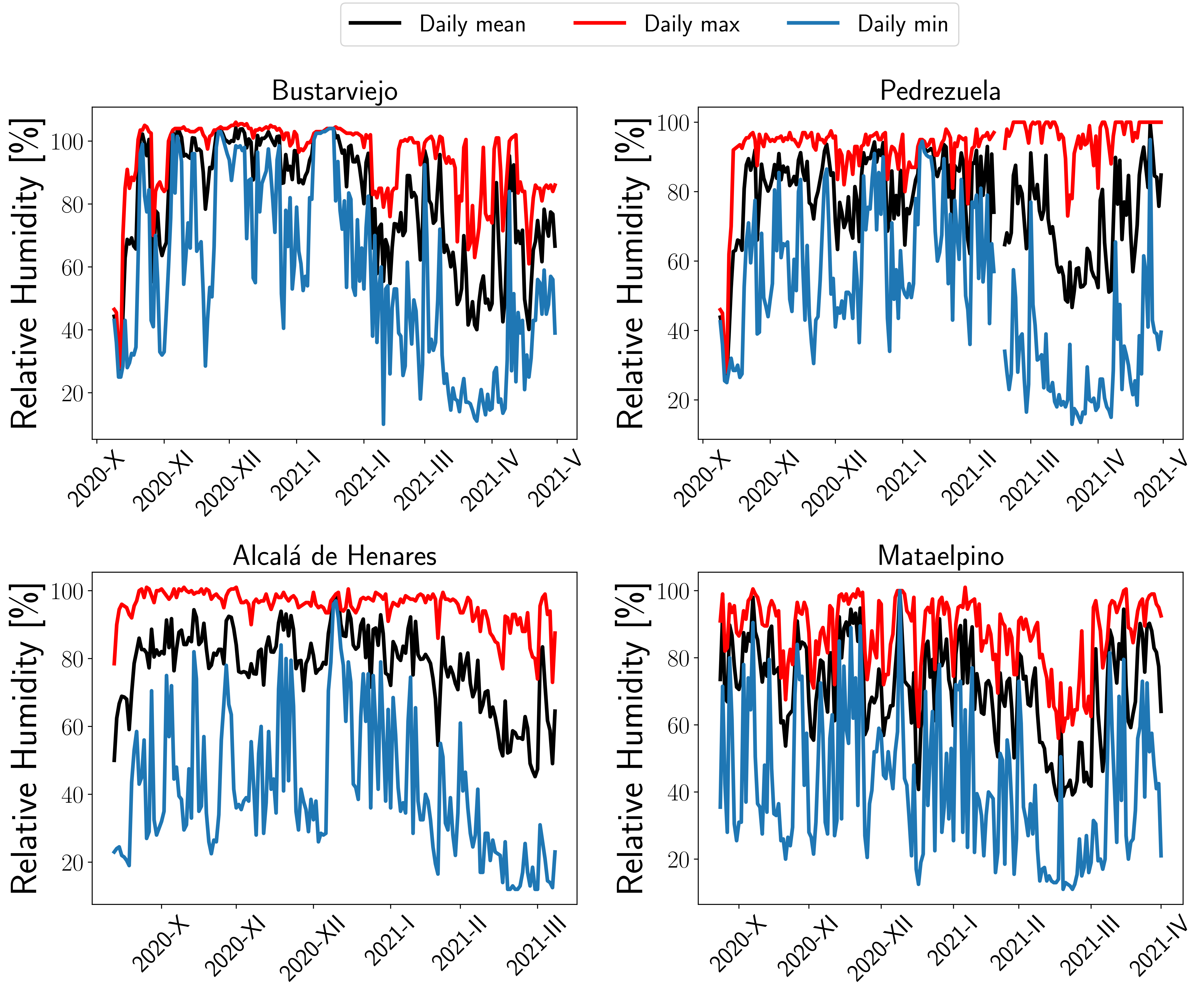

Daily mean
Daily max
Daily min
Bustarviejo
Pedrezuela
Alcalá de Henares
Mataelpino
Relative Humidity [%]
20
40
60
80
100
2020-X
2020-XI
2020-XII
2021-I
2021-II
2021-III
2021-IV
2021-V
