# Supplementary material for: Degree-day-based model to predict egg hatching of Philaenus spumarius (Hemiptera: Aphrophoridae), the main vector of Xylella fastidiosa in Europe
Source: Environ Entomol. 2023 Apr 19;52(3):350–9. doi: 10.1093/ee/nvad013 (PMC10272708; doi:10.1093/ee/nvad013)
Supplement: nvad013_suppl_Supplementary_Tables_S7_S8 [file nvad013_suppl_supplementary_tables_s7_s8.pdf]

North

1st Treatment Probability Threshold	1 Treatment Efficacy	2 Treatment efficacy (2nd at 80%)	2 Treatment efficacy (2nd at 90%)	2 Treatment efficacy (2nd at 94%)
0.2	26.7	66.7	86.7	80.0
0.2	26.7	66.7	86.7	80.0
0.2	26.7	66.7	86.7	80.0
0.3	26.7	66.7	86.7	80.0
0.3	26.7	66.7	86.7	80.0
0.3	26.7	66.7	86.7	80.0
0.3	26.7	46.7	66.7	60.0
0.3	46.7	66.7	86.7	80.0
0.4	46.7	66.7	86.7	80.0
0.4	46.7	66.7	86.7	80.0
0.4	46.7	66.7	86.7	80.0
0.4	46.7	66.7	86.7	80.0
0.4	46.7	60.0	80.0	73.3
0.5	53.3	66.7	86.7	80.0
0.5	53.3	66.7	86.7	80.0
0.5	53.3	66.7	86.7	80.0
0.5	53.3	66.7	86.7	80.0
0.5	46.7	60.0	80.0	73.3
0.6	46.7	60.0	80.0	73.3
0.6	46.7	53.3	73.3	73.3
0.6	46.7	53.3	73.3	73.3
0.6	46.7	53.3	73.3	73.3
0.6	40.0	46.7	66.7	66.7
0.7	46.7	53.3	73.3	73.3
0.7	40.0	40.0	60.0	60.0
0.7	46.7	46.7	66.7	66.7
0.7	46.7	46.7	66.7	66.7
0.7	46.7	46.7	66.7	66.7
0.8	46.7	46.7	66.7	66.7
0.8	46.7	46.7	60.0	60.0
0.8	46.7	46.7	60.0	60.0
0.8	53.3	53.3	60.0	60.0
0.8	60.0	60.0	66.7	66.7
0.9	60.0	60.0	66.7	66.7
0.9	60.0	60.0	60.0	60.0
0.9	66.7	66.7	66.7	66.7
0.9	66.7	66.7	66.7	66.7
0.9	60.0	60.0	60.0	60.0
1.0	66.7	66.7	66.7	66.7
1.0	66.7	66.7	66.7	66.7

		South		
1st Treatment Probability Threshold	1 Treatment Efficacy	2 Treatment efficacy (2nd at 80%)	2 Treatment efficacy (2nd at 90%)	2 Treatment efficacy (2nd at 94%)
0.2	30.30	66.67	69.70	66.67
0.22	36.36	69.70	75.76	72.73
0.24	39.39	69.70	75.76	72.73
0.26	39.39	66.67	75.76	72.73
0.28	42.42	69.70	78.79	75.76
0.3	42.42	69.70	78.79	75.76
0.32	42.42	66.67	75.76	72.73
0.34	42.42	63.64	72.73	72.73
0.36	42.42	63.64	72.73	72.73
0.38	45.45	63.64	72.73	72.73
0.4	45.45	60.61	69.70	69.70
0.42	51.52	66.67	75.76	75.76
0.44	51.52	63.64	72.73	72.73
0.46	54.55	66.67	75.76	75.76
0.48	54.55	66.67	75.76	75.76
0.5	51.52	63.64	72.73	72.73
0.52	51.52	63.64	72.73	72.73
0.54	51.52	63.64	72.73	72.73
0.56	51.52	60.61	69.70	69.70
0.58	48.48	57.58	66.67	66.67
0.6	48.48	54.55	63.64	63.64
0.62	51.52	57.58	66.67	66.67
0.64	54.55	57.58	66.67	66.67
0.66	57.58	60.61	69.70	69.70
0.68	57.58	60.61	69.70	69.70
0.7	51.52	54.55	63.64	63.64
0.72	51.52	54.55	63.64	63.64
0.74	51.52	51.52	60.61	60.61
0.76	51.52	51.52	60.61	60.61
0.78	51.52	51.52	60.61	60.61
0.8	54.55	54.55	63.64	63.64
0.82	51.52	51.52	60.61	60.61
0.84	51.52	51.52	57.58	57.58
0.86	51.52	51.52	57.58	57.58
0.88	54.55	54.55	57.58	57.58
0.9	54.55	54.55	54.55	54.55
0.92	48.48	48.48	48.48	48.48
0.94	45.45	45.45	45.45	45.45
0.96	42.42	42.42	42.42	42.42
0.98	33.33	33.33	33.33	33.33
